# Multiomics analysis of a resistant European turnip ECD04 during clubroot infection reveals key hub genes underlying resistance mechanism

**DOI:** 10.3389/fpls.2024.1396602

**Published:** 2024-05-23

**Authors:** Xueqing Zhou, Ting Zhong, Meixiu Wu, Qian Li, Wenlin Yu, Longcai Gan, Xianyu Xiang, Yunyun Zhang, Yaru Shi, Yuanwei Zhou, Peng Chen, Chunyu Zhang

**Affiliations:** ^1^ National Key Laboratory of Crop Genetic Improvement and College of Plant Science and Technology, Huazhong Agricultural University, Wuhan, China; ^2^ College of Agronomy and Biotechnology, Yunnan Agricultural University, Kunming, China; ^3^ Industrial Crops Research Institute, Yunnan Academy of Agricultural Sciences, Kunming, China; ^4^ Rice and Oil Research Institute, Yichang Academy of Agricultural Science, Yichang, China

**Keywords:** European fodder turnip, *Plasmodiophora brassicae*, transcriptome, miRNA, degradome, *APS4*

## Abstract

The clubroot disease has become a worldwide threat for crucifer crop production, due to its soil-borne nature and difficulty to eradicate completely from contaminated field. In this study we used an elite resistant European fodder turnip ECD04 and investigated its resistance mechanism using transcriptome, sRNA-seq, degradome and gene editing. A total of 1751 DEGs were identified from three time points after infection, among which 7 hub genes including *XTH23* for cell wall assembly and two *CPK28* genes in PTI pathways. On microRNA, we identified 17 DEMs and predicted 15 miRNA-target pairs (DEM-DEG). We validated two pairs (miR395-*APS4* and miR160-*ARF*) by degradome sequencing. We investigated the miR395-*APS4* pair by CRISPR-Cas9 mediated gene editing, the result showed that knocking-out *APS4* could lead to elevated clubroot resistance in *B. napus*. In summary, the data acquired on transcriptional response and microRNA as well as target genes provide future direction especially gene candidates for genetic improvement of clubroot resistance on Brassica species.

## 1 Introduction

The *Brassica* genus include many economically important crop species such as cabbage, turnip and oilseed rape (Cheng et al., 2016). Among those, rapeseed is particular since its seeds are of high nutrient value and balanced protein composition, high oil contents as an industrial oil source, and also for the residue left from seed oil extraction as feeding proteins (Chmielewska et al., 2021). During recent years, the oilseed production is greatly influenced by the outbreak of clubroot disease, which is caused by a soil-borne pathogen *Plasmodiophora brassicae*. *P. brassicae* belongs to Endomyxa and it has host specificity towards crucifer plants only (Dixon, 2009). The infection starts from root and ultimately cause formation of root galls and dysfunction on water and nutrient transport from root to aboveground part, therefore causing plant wilting and even death. Severe disease causes great loss of yield production that range from 20-30% up to 100% (Chai et al., 2014; Strehlow et al., 2015). In China each year about 3.2-4.0 million hectares are affected by clubroot. The resting spore of *P. brassicae* can survive up to 20 years in the soil, therefore once a field is contaminated it is almost impossible to eradicate the pathogen. Therefore, the most cost-effective way to prevent the disease from spreading is breeding and cultivation of resistant cultivars (Neik et al., 2017).

Most of the clubroot resistant (CR) loci are isolated from *B. rapa*, *B. oleracea* and mostly from the A genome. Some of the identified CR loci include *PbBa1.1* and *Crr2* from A01chromosome (Suwabe et al., 2003; Chen et al., 2013), *CRc* and *Rcr8* from A02 chromosome, and the maximum number of 16 from the A03 chromosome [*PbBa3.1* (Chen et al., 2013), *PbBa3.2* (Chen et al., 2013), *Crr3* (Hirai et al., 2004), *CRk* (Sakamoto et al., 2008), *CRd* (Pang et al., 2018), *PbBa3.3* (Chen et al., 2013), *BraA.CR.a* (Hirani et al., 2018), *BraA3P5X.CRa/bKato1.1* (Fredua-Agyeman et al., 2020), *Rcr1* (Chu et al., 2014), *CRa* (Matsumoto et al., 1998), *Rcr2* (Huang et al., 2017), *BraA3P5X.CRa/bKato1.2* (Fredua-Agyeman et al., 2020), *BraA.CR.c* (Hirani et al., 2018), *CRb* (Piao et al., 2004), *Rcr4* (Yu et al., 2017), *Rcr5* (Huang et al., 2017)]. In addition, *CrrA* on A05 and *Crr4* on A06 chromosome, and the second number of CR loci on A08 chromosome [*PbBa8.1* (Chen et al., 2013), *Rcr3* (Karim et al., 2020), *Rcr9wa* (Karim et al., 2020), *BraA.CR.b* (Hirani et al., 2018), *Rcr9* (Yu et al., 2017), *Crr1* (Suwabe et al., 2003), *CRs* (Laila et al., 2019), *CRA8.1* (Wang et al., 2022b)]. Since many of these CR loci are allelic but of different source, a recent study integrated all 28 loci reported from the A genome and mapped them into 15 CR loci on a resistant reference genome from ECD04 (Yang et al., 2022a). Based on the gene annotation, 62 NLR-type candidate R genes were proposed. Besides NLR, a pericycle-expressed ion channel protein WeiTsing was also recently identified, that confers broad-spectrum resistance to *P. brassicae* (Wang et al., 2023). These genes and their homologs serves as good candidate gene resource for potential use in resistance breeding.

So far, there are three CR genes that have been cloned and functionally validated, including *Crr1a* and *CRA8.2.4* from A08, and *CRa* from A03 chromosome. All three genes encode NLR proteins (Wu et al., 2013; Hatakeyama et al., 2017). The NLR proteins can sense the presence of pathogen effectors to induce the ETI (effector triggered immunity) pathways in plant-pathogen recognition (Wang et al., 2019). The two plant immune pathways, PTI (pattern-triggered-immunity) and ETI are unique both in epitope recognition and time/stage of immune response, but they are also interconnected such as the effectors secreted from pathogen into plant host cell can suppress PTI response while NLR recognition on specific effectors can trigger ETI response to reprogram defense gene expression (Dangl et al., 2013; Song et al., 2020). However, also because of the specificity between the pathogen effector and host NLR receptors, the resistance mediated by a single R gene might be lost due to the arising mutations and eventually failure of recognition. In order to deal with the ongoing selection pressure and risk for losing resistance, understanding the resistance mechanism and the molecular details between clubroot pathogen and host plant would be very helpful for breeding work and developing resistant cultivars using different R genes.

Plant microRNAs are encoded by endogenous MIR genes, most of which are in-between coding genes with minority residing in introns in protein-coding genes (Rajagopalan et al., 2006; Yan et al., 2012; Yang et al., 2012). Most miRNAs are 21 nt, less common as 20 or 22nt, rare cases for 23 or 24 nt (Wu et al., 2010; Montes et al., 2014; Axtell and Meyers, 2018). The mature miRNA is loaded onto the miRISC (miRNA-induced silencing complex), they can mediate cleavage or translational repression on target mRNA by partial complementarity, therefore downregulate target gene expression (Song et al., 2019; Cui et al., 2020). Numerous studies have illustrated the important roles of miRNAs on plant immunity (Navarro, 2016). For example miR393, the first microRNA identified to participate in PTI response in *Arabidopsis*, can be induced by flagellin-derived peptide and target *TIR1*, *AFB2* and *AFB3* to suppress auxin signaling pathway to inhibit the growth of *Pseudomonas* pathogen. On ETI pathways, people found miR1885 as negative regulator of *BraTNL1* which is a TIR-type NLR protein that confers antiviral immunity (Cui et al., 2020). Another study showed that six antagonistic miRNA-target pairs were involved in response to clubroot pathogen infection in a resistant *B. napus* material 409R (Li et al., 2021). Many miRNA-target pairs participate in plant immune response, such as miR169-*NF-YA* in bacterial plant diseases *Ralstonia solanacearum* (Hanemian et al., 2016), miR164-*NAC4* in HR or PCD response in *Arabidopsis* (Lee et al., 2017), and host miRNA export to silence fungi genes for disease resistance in cotton (Zhang et al., 2016). Studies in rice revealed that miR395-*OsAPS1* modulates sulfate metabolism to promotes resistance (Yang et al., 2022b).

Although numerous studies have shown molecular mechanism behind host-pathogen interaction in different diseases, how miRNA participate in clubroot resistance in crucifer plant such as the European turnip ECD04, which showed superior resistance to many races of clubroot pathogen, is currently unknown. Not only microRNAs, but also the targets, and how they influence disease outcome, is unclear. In this study, we used ECD04 and clubroot inoculation to investigate the transcriptome, sRNA-seq and degradome sequencing, to identify key microRNAs and their targets during infection course. Our work shed light on the interaction between key microRNAs and their targets leading to clubroot resistance in European turnip ECD04.

## 2 Materials and methods

### 2.1 Plant materials and clubroot resistance evaluation

For the clubroot resistance study, we used *B. rapa* (ECD04) derived from the European Clubroot Differential Set, which genome has been completed assembled in our previous study (Yang et al., 2022a). ECD04 roots were inoculated with *P. brassicae* strain collected from Zhijiang (Hubei, China) (Chen et al., 2016), root sample treated with water was used as mock. In detail, the collected galls were thawed at room temperature, mixed with buffer, crushed and filtered, and the spore concentration was diluted to 10^7^ resting spores per milliliter in sterile distilled water used for the infection experiments. The germinated seeds were sown into the soil and after 7 days 1 ml of spore suspension was applied to their roots. Samples were collected at 18, 24, or 30 days in triplicates, resulting in 18 cDNA libraries including I18, I24 and I30 (inoculated), and M18, M24 and M30 (mock). Total RNA was extracted using TRIzol (Invitrogen Co Ltd, USA) according to the manufacturer’s instructions. The same set of samples were used for RNA-seq and sRNA-seq.

At about 35 days after inoculation, disease symptoms can be classified as 0-3 grade based on their root morphology, roots with no gall were classified as grade 0, lateral roots with small galls were classified as grade 1, lateral roots with large galls were classified as grade 2, and main roots with large galls were classified as grade 3. Based on the number of individual plants in different disease classes, the disease index (DI) of the material was calculated to assess the disease resistance of the material (Strelkov et al., 2006).


$$\text{DI}\left(\%\right)=\frac{\sum \left(n\times 0+n\times 1+n\times 2+n\times 3\right)}{N\times 3}\times 100\%$$

Where *n* is the number of plants in each grade; *N* is the total number of plants; and 0, 1, 2, and 3 are the disease symptom grade.

### 2.2 Transcriptome sequencing and pre-processing

cDNA library preparation and sequencing were performed using Hifair II First Strand cDNA Synthesis Kit (Beijing Genome Institute, China). Messager RNA was isolated using oligo (dT) primers, fragmented and cDNA was synthesized with random primers. Second-strand cDNA was synthesized using buffer, deoxyribonucleotide triphosphates (dNTPs), RNase H, and DNA polymerase I. The synthetic cDNA was subjected to end repair by polymerase with Seloxa adapters, and suitable fragments were retrieved by agarose gel electrophoresis. Polymerase chain reaction (PCR) amplification was carried out to enrich the purified fragments. RNA-Seq was carried out using SEQ-500 platform (BGI, China).

### 2.3 Identification and expression analysis of differential expression genes

Gene expression levels were analyzed by R v3.6.2 package “DESeq2” (1.10.1). DEGs were identified based on 1) adjusted P<0.05, 2) Fragments Per Kilobase of transcript per Million mapped reads (FPKM) fold change ≥2.0. Function annotation of the DEGs were retrieved from known databases (Yang et al., 2022a). The Gene Ontology (GO) and KEGG (Kyoto Encyclopedia of Genes and Genomes) enrichment analysis of all DEGs were performed using the “TBtools” software package (Chen et al., 2020). The cut-off value for GO and KEGG terms were p-value< 0.05. Weighted gene co-expression network analysis was implemented with the WGCNA package in R (Langfelder and Horvath, 2008). Weighted networks with scale-free topology was constructed using a soft threshold power of 9 with R^2^-value > 0.84. Heatmap and module clustering were used to visualize the co-expression network of all DEGs. Correlations between different modules and conditions (Inoculation vs. Mock) were calculated, selected modules were visualized by cytoscape software (Michael et al., 2010).

### 2.4 Small RNA sequencing and expression analysis of miRNA

Small RNAs in the size range of 18-30 nt were gel purified and ligated to adapters and reverse transcribed to cDNA, then PCR amplification was carried out to enrich the purified fragments. sRNA-seq was carried using SEQ-500 platform (BGI, China). Raw reads obtained from sequencing were processed for quality control and low-quality reads were removed, reads were trimmed and filtered for primer contamination and poly A tail using Trimmomatic v0.35. Reads shorter than 18 nt and longer than 30 nt were discarded. The clean reads were further screened against tRNA, rRNA, snRNA sequences, redundant reads were combined into unique sequences for miRNA prediction. Unique reads were mapped to plant miRNAs from miRbase (https://www.mirbase.org/) to identify known miRNAs. The alignment was done using blastn and these unique reads were also mapped to the ECD04 genome using bowtie2 (Langmead and Salzberg, 2012). For the identified precursor, novel miRNAs were identified using mirDeep2 (https://github.com/rajewsky-lab/mirdeep2). The expression of miRNAs was determined by the R package “DESeq2”. DEMs were identified by 1) adjusted P<0.05, 2) transcript per million mapped (TPM) fold change ≥2.0.

### 2.5 Degradome sequencing and data processing

We used only samples at 24 dpi (I24 and M24) for degradome sequencing. The purified cDNA library was sequenced on an Illumina HiSeq 2000 instrument. After removing low-quality data, further mapped to the *B. rapa* genome to obtain cDNA sense and antisense tags. The tags that mapped to cDNA or mRNA sequences were used for cleavage sites prediction by CleaveLand (Addo-Quaye et al., 2009), categories definition (containing 0–4 categories, 0 for best match) and T-plot figures. psRNATarget was used to predict miRNA targets (Dai et al., 2018).

### 2.6 Quantitative real-time PCR analysis

To validate the RNA-seq and sRNA-seq results, 6 DEGs and 3 DEMs were subjected to qRT-PCR (**Supplementary Table 9**). *Bra-UBC10* and *Bra-U6* were used as reference genes, respectively. Total RNA was extracted from the same samples used for RNA-seq or sRNA-seq. mRNA quantification was performed with HiScript II Q RT SuperMix for qPCR Kit (Vazyme Co Ltd, China) and ChamQ Universal SYBR qPCR Master Mix Kit (Vazyme, China). MiRNA quantification was carried out by miRcute Plus miRNA First-Strand cDNA Synthesis Kit (TIANGEN Co Ltd, China) and miRcute Plus miRNA qPCR Detection Kit (TIANGEN, China). CFX384 Touch Real-Time PCR Detection System (Bio-Rad Co Ltd, USA) was used for qRT-PCR.

### 2.7 Generation of CRISPR/Cas9 construct and transgenic plants

To generate the *Bra-APS4* CRISPR/Cas9 constructs, one 20bp sgRNA targeting two positions, one targeting the first exon of *BnaA06G0440400ZS* and the other targeting the first exon of *BnaC07G0222000ZS*, was cloned into the pYLCRISPR/Cas9 expression vector (Ma et al., 2015). These constructs were transferred into *Agrobacterium tumefaciens* strain GV3101 (Tsingke Co Ltd, China) and transformed to *B. napus* 409S by hypocotyl transformation. Disease phenotypes were investigated by pot inoculation with *Pb*Zj (Zhijiang, Hubei, China) pathogen, using 409R and 409S as resistant and susceptible controls, respectively. 409S and 409R is a pair of NILs, 409R contained clubroot resistance locus from Chinese cabbage material (Li et al., 2021).

## 3 Results

### 3.1 Transcriptional dynamics in ECD04 upon *P. brassicae* infection

**Figure 1:**
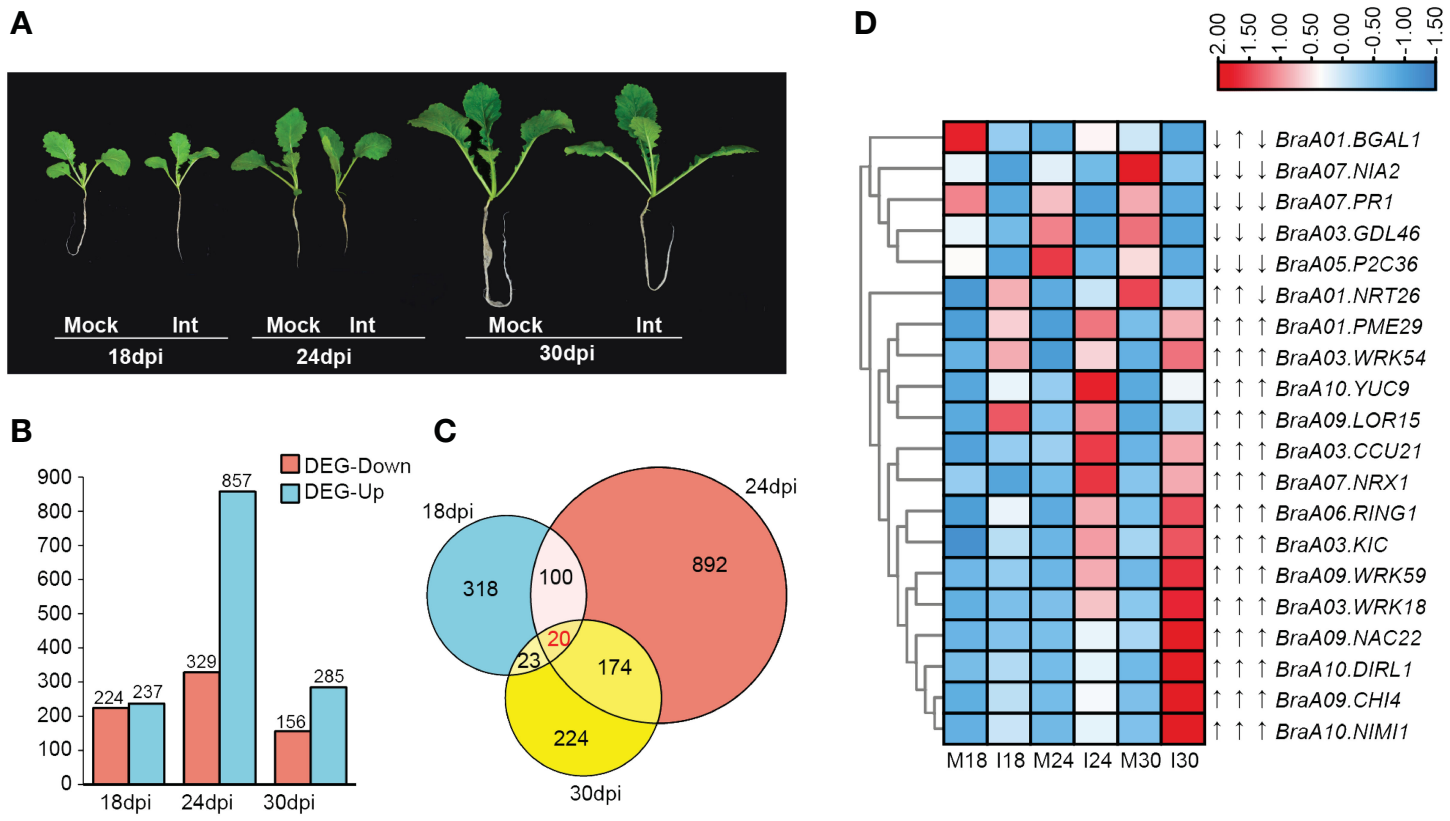
An overview of differentially expressed genes (DEGs) in ECD04 upon *P. brassicae* infection. **(A)** Phenotypes of ECD04 after inoculation with *Pb*Zj at different time points. **(B)** Number of DEGs in ECD04 at 18th, 24th and 30th day post inoculation (dpi). **(C)** Venn diagram analyses of the total numbers of DEGs between 18 dpi, 24 dpi, and 30 dpi. **(D)** Heatmaps showed the FPKM of common differential expression genes at three time points.

ECD04 is a resistant material that showed broad resistance to different races of clubroot pathogen *P. brassicae* (Yang et al., 2022a). We performed RNA-seq to analyze global transcriptome changes in order to explore its defense response at different stages of *P. brassicae* infection (**Figure 1**). We chose three time points (18 dpi, 24 dpi and 30 dpi, dpi=days post infection) and un-infected plants as mock control. RNA-seq samples created 40-43 million raw reads and 39-42 million clean reads with good quality (**Supplementary Table 1**). On average, 98% of clean data were mapped to the ECD04 reference genome, among which 93% were uniquely matched (**Supplementary Table 1**). These transcriptome data were used for further analysis.

Differential gene expression was made first pairwise at each time points between the mock group (M) and the inoculation (I) group (**Figures 1B, C**), i.e. M18 vs. I18, M24 vs. I24, and M30 vs. I30. As shown in **Figure 1B**, 461 (237 up and 224 down), 1186 (857 up and 329 down), and 441 (285 up and 156 down) DEGs (differential expressed genes) were detected at 18 dpi, 24 dpi and 30 dpi, respectively, after overlapping there are 1751 DEGs with threshold of |log2FoldChange| ≥ 1, and Padj< 0.05 (**Figure 1B**; **Supplementary Table 2**). Compared to 18 dpi and 30 dpi, 24 dpi had the most DEG, and more genes were found to be up-regulated then down-regulated (**Figure 1B**). As shown on Venn diagram, a small fraction of DEGs was shared at different time points, 20 genes were differentially expressed at three time points (**Figure 1C**). Moreover, among these 20 common DEGs, 13 were sustainable up-regulated from 18 dpi to 30 dpi, including those involved in SAR (*NIMI1*, *KIC*, *PME29*, *WRK*, *CHI4*) and one is down-regulated (*PR1*) (**Figure 1D**). In particular, there are 3 DEGs that showed different trends, including Beta-galactosidase 1 (*BGAL1*) with “down-up-down” for 18-24-30 dpi in between mock and inoculation samples, *NRX1* with “down-up-up” for 18-24-30 dpi, and *NRT26* with “down-down-up”, respectively (**Figure 1D**). Most of these common 20 DEGs are previously shown to be associated with pathogen infection. Overall, these results indicate stage- specific response upon *P. brassicae* infection in ECD04.

### 3.2 Functional enrichment analyses of differentially expressed genes

**Figure 2:**
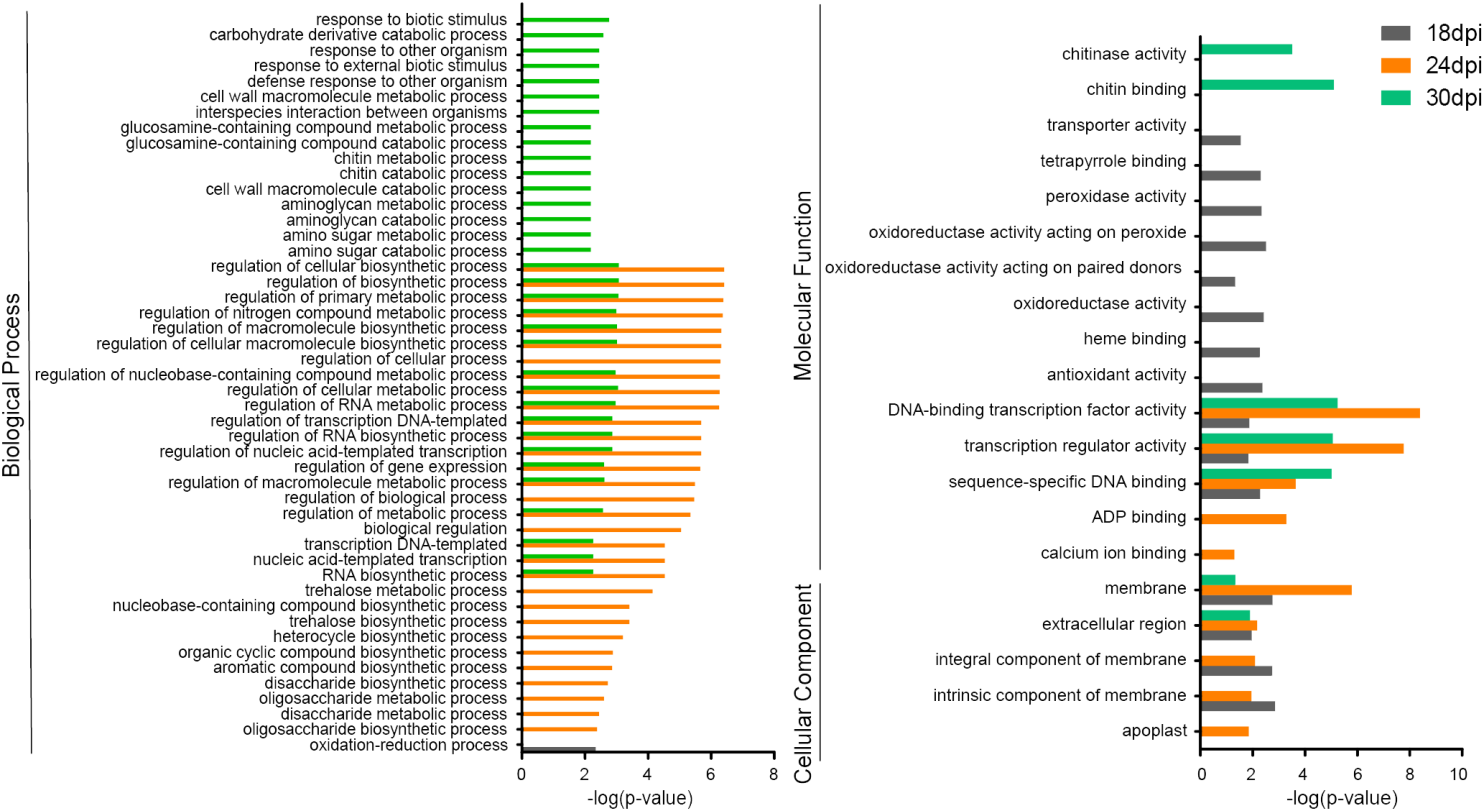
Gene Ontology (GO) pathway assignments for DEGs at three time points, separately.

GO enrichment was performed on 1751 DEGs identified for all three time points between mock and inoculation samples (**Supplementary Table 2**). Since the Biological process is enriched to more terms, we set the value of p-value to 0.01. A total of 68 GO terms were identified in three categories: biological process (BP, 48 terms), molecular function (MF, 15 terms), cellular component (CC, 5 terms) (**Figure 2**). In particular, the 461 DEGs at 18 dpi were assigned to 16 terms (BP, 1 term; MF, 11 terms; and CC, 4 terms. Likewise, 1186 DEGs at 24 dpi were assigned to 46 terms (BP 35 terms, MF 5 terms, and CC 5 terms). 441 DEGs at 30 dpi were assigned to 52 terms (BP 44 terms, MF 5 terms, and CC 2 terms) (**Figure 2**).

DEGs from 18 dpi specially enriched in oxidation-reduction process, oxidoreductase activity, and antioxidant activity (**Figure 2**). 24 dpi always show higher p-value than other times point, like DNA-binding transcription factor activity and membrane, and it specially enriched in calcium ion binding. And 30 dpi was specially enriched in response to biotic stimulus and chitin binding (**Figure 2**). The common pathways identified for DEGs for all three time points includes the following: DNA-binding transcription factor activity (MF term), membrane (CC term) and extracellular region (CC term) (**Figure 2**). These results suggest a dynamic change in resistant *B. rapa* material ECD04 upon *P. brassicae* infection that involves genes that play crucial roles in clubroot resistance in different pathways.

### 3.3 Co-expression network analysis identifies hub genes associated with clubroot resistance

**Figure 3:**
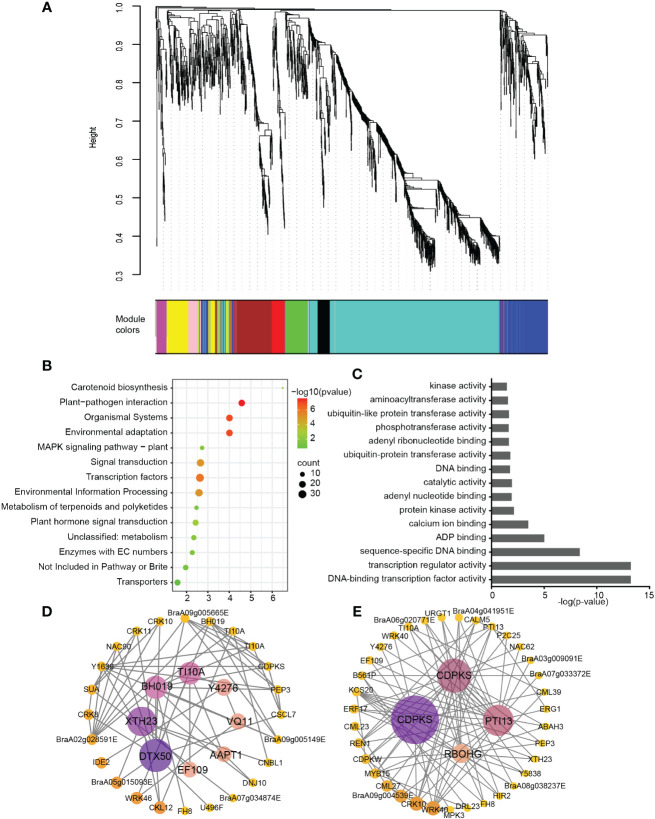
Weighted gene co-expression network analysis of relative differentially expressed genes. **(A)** Hierarchical cluster dendrogram showed co-expression modules. Each leaf (short vertical line) in the tree represented one gene. The genes were clustered based on dissimilarity measure. The major tree branches, corresponded with the color rows below the dendrogram, constituted the modules. **(B)** KEGG enrichment of “turquoise” module genes. **(C)** GO enrichment of “turquoise” module genes. **(D)** Interaction network of the identified hub genes in “turquoise” module. **(E)** Interaction network of “Plant-pathogen interaction” in “turquoise” module.

Defense response involves substantial transcriptional reprogramming in plants. Weighted gene coexpression network analysis (WGCNA) was performed to understand the correlation between the sample expression profile and the treatment. A scale-free WGCNA network was constructed with the soft threshold power of 20 with threshold R^2^ > 0.84 (**Figure 3A**). Out of the total 1751 DEGs, 1283 were assigned into 10 distinct modules (**Figure 3A**; **Supplementary Figure 1**). Each module represents a cluster of highly interconnected genes with similar expression changes.

KEGG pathway analysis were performed on the genes in these modules, we found only “blue”, “brown”, “black” and “turquoise” showed significant enrichment with p-value< 0.05 (**Supplementary Table 3**). The genes in “blue” module was mostly enriched in glutathione metabolism, the “brown” module genes was mostly enriched in glycolysis/gluconeogenesis (**Supplementary Table 3**). Based on the correlation values in inoculation samples between 18 dpi, 24 dpi and 30 dpi time points (**Supplementary Figure 1**), we chose the “turquoise” module for KEGG, GO and gene-interaction network construction (**Figures 3B-E**). The “turquoise” module contained a total of 781 genes, The gene-interaction network constructed by Cytoscape of turquoise module showed hub genes including *DTX50* (detoxification, transporter), *XTH23* (xyloglucan endoglucosylase/hydrolase), *BH019* (Transcription factor), *TIFY10A* (repressor of jasmonate responses) that might be important for *P. brassicae* defense response. In turquoise module, the most significantly enriched KEGG pathway was plant-pathogen interaction (**Figure 3B**), and the most significantly enriched GO pathway was DNA-binding transcription factor activity and transcription regulator activity (**Figure 3C**). Furthermore, the interaction network of genes in KEGG term associated with “plant-pathogen interaction” showed additionally two of *CDPKS* (Calcium-dependent protein kinase 28) and *PTI13* (PTI1-like tyrosine-protein kinase 3) during this process. In total, there are 7 gene were brought out after WGCNA and interaction network analysis, they might be the hub genes for clubroot resistance in ECD04 (**Figures 3D, E**).

### 3.4 MiRNA dynamics in ECD04 after *Plasmodiophora* inoculation

**Figure 4:**
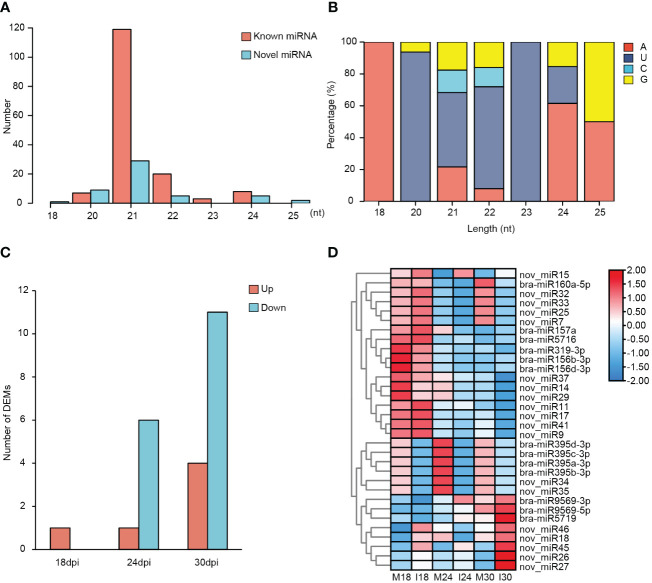
Identification and characterization of miRNAs in ECD04 upon *P. brassicae* infection. **(A)** Length distribution of miRNAs. **(B)** Preference of the first nucleotide for miRNAs. **(C)** Number of DEMs in ECD04 at 18, 24 and 30 dpi. **(D)** Heatmap of differentially expressed miRNAs. M18, mock 18 dpi; I18, inoculation 18 dpi; M24, mock 24 dpi; I24, inoculation 24 dpi; M30, mock 30 dpi; I30, inoculation 30 dpi.

To investigate the regulation between genes and miRNAs in disease-resistant turnip ECD04, the same set of libraries mentioned above were used for sRNA-seq to capture micro RNA expression changes (**Figure 4**). Above 21.5 million reads were obtained on average, after comparing with rRNA, snRNA, tRNA, 15 million filtered reads were used for miRNA annotation (**Supplementary Table 1**). A filtering pipeline was used to identify first the known miRNAs. 157 known miRNA were obtained from the 18 sRNA libraries deposited in miRBase. Besides, 51 potential novel miRNA candidates were identified by mirDeep (**Supplementary Table 4**). The length of known miRNAs and novel miRNAs ranged from 18-25 nt (**Figure 4A**), the majority of 23 nt long miRNAs were characterized by a 5’-uridine (**Figure 4B**). 5’-uridine has been observed in many plant species and is a characteristic attribute of DCL1 cleavage and AGO1 association (Reinhart et al., 2002; Rajagopalan et al., 2006). On the other hand, the 157 known miRNAs were classified into 60 miRNA families, with miR156 being the largest family with 14 members (**Supplementary Figure 2**).

According to the TPM values of these miRNAs, we identified DEMs (different expressed miRNAs) at 18, 24 and 30 dpi in between mock and inoculation conditions (**Figure 4C**; **Supplementary Table 5**). DEMs were selected based on p-value< 0.05 and |fold change| > 2. As result, 2, 4 and 8 DEMs were found at 18dpi, 24dpi and 30 dpi, respectively (**Figure 4C**). Further, to interrogate the miRNAs involve in *P. brassicae* resistance, we looked into DEMs that exhibited similar patterns at all three times points. Indeed, 1, 1 and 4 miRNAs were constantly up-regulated at 18, 24 and 30 dpi, respectively; meanwhile 6 and 11 miRNAs were down-regulated at 24 and 30 dpi, respectively (**Figure 4D**). Notably, bra-miR395a/b/c/d-3p and two novel miRNAs (nov_miR34/35, also members of MIR395) were down-regulated both at 24 dpi and 30 dpi, with more significant downregulation at 24dpi (**Figure 4D**). Overall, these results indicate stage-specific regulation on miRNA expression as well in ECD04 upon *P. brassicae* infection, possibly leading to differential expression of downstream target genes.

### 3.5 Degradome sequencing and identification of miRNA-target pairs

Plant miRNAs usually have perfect or near-perfect complementarity with their targets. To investigate the targets of miRNAs identified above, we used degradome sequencing and generated 22 and 31 million reads for Mock and Inoculation samples, respectively (**Supplementary Tables 6, 7**). After filtering, 21.6 and 30.5 million reads were obtained, and 108 and 93 miRNA target pairs were identified for mock and inoculation sample, respectively (**Supplementary Tables 6, 7**). The maximum cleavage sites belonged to category 0 (average 62%) and minimum to category 4 (average 6%). After filtering with category< 2, 90 and 81 miRNA target pairs were left for mock and inoculation conditions. In total, 128 miRNA-target pairs were identified from degradome sequencing (**Supplementary Tables 6, 7**). Annotation of the target genes suggests most to be disease resistance genes like NBS-LRR, and TFs like auxin response factor (*ARF*) and scarecrow-like protein (*SCL*).

As for target genes, 61 targets were detected both in mock and inoculation conditions, whereas 44 and 31 were only for mock or inoculation (**Supplementary Tables 6, 7**). The maximum number of targets were obtained for miR1885 family, where 20 targets were identified and most of them were NBS-LRR genes (**Supplementary Tables 6, 7**). The second group of targets was related to plant hormone signal transduction, like bra-miR160/bra-miR167/nov_miR39 and their target *ARF*, bra-miR171 and its target encoding scarecrow-like protein, bra-miR172 and its target *RAP*/*TOE2*/*AP1* etc (**Supplementary Tables 6, 7**). Other miRNA-target pairs involve different metabolic pathways, such as bra-miR164 and target *NAC*, bra-miR168 with target *AGO1* and bra-miR162 with target *DCL1* for RNA-mediated gene silencing, and miR395 with its target *APS4* (ATP sulfurylase 4) for sulfur metabolism (**Supplementary Tables 6, 7**).

**Figure 5:**
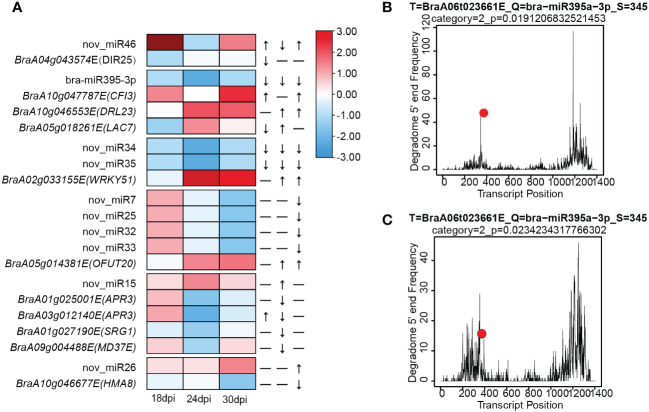
MiRNA-Target pairs involved in resistance to *P. brassicae*. **(A)** A combined view of the expression level of selected coherent pairs of differentially expressed miRNAs and their predicted target genes. **(B, C)** Degradome sequencing of Bra-miR395a-3p with its target *APS4* (*BraA06t023661E*) in mock **(B)** or inoculation **(C)** conditions.

Combing with DEMs to transcript levels of target genes in response to *P. brassicae*, only bra-miR395a, bra-miR160a and nov_miR7 were detected in degradome sequencing. Bra-miR395a was down-regulated in 24/30 dpi and its target, *BraA06g023661E* (*APS4*) although not DEG, was up-regulated at 24 dpi. The cleavage of *Bra-APS4* by bra-miR395a was confirmed by degradome sequencing (**Figures 5B, C**). Bra-miR160a was down-regulated in 30 dpi but its target are not DGEs, among its targets, *BraA07g035249E* (*ARF*) and *BraA02g030057* (*BAG1*) were slight up-regulated in 30 dpi. As for nov_miR7, although this miRNA was down-regulated in DEM, but its predicted targets *BraA07g034603E* and *BraA09g004346E* (*FBX6*) could not be detected in RNA sequencing, probably due to too low expression level. Finally, we used psRNATarget server and predicted a total of 2319 miRNA-target pairs involving 208 miRNAs (157 known and 51 novel). KEGG enrichment on the target genes identified terms in Monobactam biosynthesis, Sulfur metabolism and Selenocompound metabolism (**Supplementary Table 8**). The expression of both *P. brassicae* responsive miRNAs (from sRNA-seq) and their targets (from RNA-seq) were integrated and inferred 15 critical miRNA-target pairs during *P. brassicae* infection (**Table 1**, cutoff value p< 0.05 and |log_2_FC| > 1). Among these, 1, 5 and 6 pair were identified from 18 dpi, 24 dpi and 30 dpi, respectively (**Table 1**). Three pairs were identified both for 24 and 30 dpi, which involved potential disease resistance protein (*DRL23*) and Transcription factor (*WRKY51*) (**Table 1**, **Figure 5A**).

**Table 1:** DEMs-DEGs pairs responding to *P. brassicae* identified by psRNATarget.

miRNA	Time point	log_2_FC	Target Transcript	Time point	log_2_FC	Annotation
nov_miR46	18dpi	13.11	BraA04t043574E	18dpi	-1.03	DIR25
bra-miR395a-3p*	24/30dpi	-2.28/-1.11	BraA05t018261E*	24dpi	1.35	LAC7
			BraA10t047787E	30dpi	2.47	CFI3
			BraA10t046553E	24/30dpi	2.04/1.88	DRL23
nov_miR34/35	24/30dpi	-2.88/-2.06	BraA02t033155E*	24/30dpi	5.40	WRKY51
nov_miR32	30dpi	-1.51	BraA05t014381E	24/30dpi	1.43/1.61	OFUT20
nov_miR25 nov_miR7 nov_miR33		-1.56				
nov_miR15*	24dpi	1.4	BraA01t025001E*	24dpi	-1.63	APR3
			BraA03t012140E*	24dpi	-2.16	APR3
			BraA01t027190E*	24dpi	-1.36	SRG1
			BraA09t004488E	24dpi	-1.19	MD37E
nov_miR26*	30dpi	1.44	BraA10t046677E	30dpi	-1.53	HMA8

*Bold lettered mRNAs and miRNA were experimentally verified by qRT-PCR.

### 3.6 qRT-PCR validation of significant DE miRNAs and genes

To validation the results from sRNA-seq and RNA-seq for the selected miRNA-target pairs, we selected 5 DEGs and 3 DEMs from the miRNA-target interaction network, and 1 gene (*BraA07g033342E*) that was shared as DEG for all three time points (**Figure 1C**, **Table 1**). The qRT-PCR results were highly consistent with the results from RNA-Seq/sRNA-Seq data for levels of these mRNAs/miRNAs, indicating the inferred miRNA-target interaction to be reliable and might be important during the defense response upon clubroot infection (**Supplementary Figure 3**).

### 3.7 Knocking-out *APS4* could lead to elevated clubroot resistance in *B. napus*

**Figure 6:**
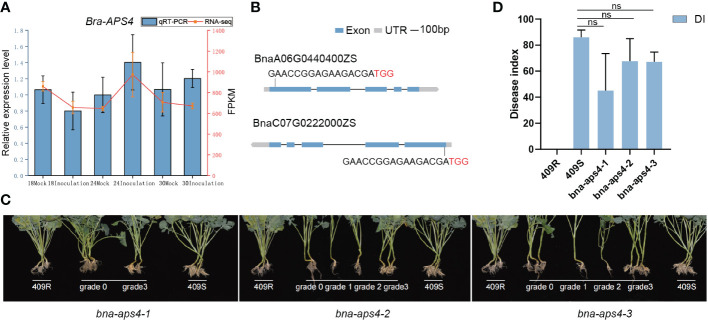
Phenotype of *bna-aps4* mutants. **(A)** Transcription level of *APS4* by RNA-seq and qRT-PCR. **(B)** The target site designed for knocking out the *Bna-APS4* gene by the CRISPR/Cas9 strategy. The target site nucleotides are shown in capital letters, and the PAM site is indicated as red capital letters. **(C)** Disease phenotype of bna-aps4 mutants after inoculation with *Pb*Zj. Grade 0, 1, 2, 3 represent different disease level, 409R and 409S were used as resistant and susceptible control, respectively. **(D)** Disease index of *bna-aps4* mutants upon *Pb*Zj infection. ns indicate insignificance.

Based on the correlation analysis of miRNAs expression and degradome sequencing, we identified bra-miR395a to be associated with *P. brassicae* infection, which indeed the members of the same microRNA family has been reported in previous studies (Li et al., 2021). Bra-miR395a targets *BraA06g023661E* (*APS4)*, which encodes for a protein for sulfur transfer (Hatzfeld et al., 2000). *Bra-APS4* transcript levels were slightly up-regulated at 24 dpi and 30 dpi during *P. brassicae* infection (**Figure 6A**). Since ECD04 is difficult to transform, we checked its homologs in *Brassica napus* ZS11, and found the *Bna-APS4* has two copies (*BnaA06G0440400ZS* and *BnaC07G0222000ZS*). In order to investigate the role of *APS4* for clubroot resistance, we used CRISPR/Cas9 to knock out the two copies of *Bna-APS4* in a susceptible *B. napus* material 409S, using one sgRNAs specific for *BnaA06G0440400ZS* and *BnaC07G0222000ZS*, respectively (**Figure 6B**). Three individual edited mutants were obtained, all leading to *Bna-APS4* loss-of-function (**Supplementary Figure 4**). When disease phenotypes were examined upon *Pb*Zj infection, the DI of resistant and susceptible control and three independent mutant lines were 0, 86, 45, 68, 67, respectively (**Figures 6C, D**). The lowest DI was observed in *bna-aps4-1*, shown by less gall formation on lateral and main roots at 35 dpi compared to susceptible control 409S (**Figure 6C**). Therefore, knocking out *Bna-APS4* in 409S background changed disease phenotype from very susceptible to a moderate susceptible/more resistant phenotype, suggesting a functional *Bna-APS4* gene might lead to a less resistance phenotype, i.e. *Bna-APS4* may serve as a negative regulator of clubroot resistance in *Brassica napus*.

## 4 Discussion

The clubroot disease has now become a serious threat for rapeseed and other cruciferous crop cultivation worldwide. Developing resistant cultivars requires the fundamental understanding of the molecular mechanism behind clubroot resistance, in order to create crop species with higher and broader resistance. The technical advance on Hi-throughput sequencing facilitates the possibility to understand the basic genomic information of ECD04 with superior resistance towards different races of clubroot pathogens. Yet, compared to other crops, studies on clubroot resistance in *B. rapa* are still rather limited (Chen et al., 2016).

### 4.1 Transcriptome analysis can provide hub genes for resistance mechanism

**Figure 7:**
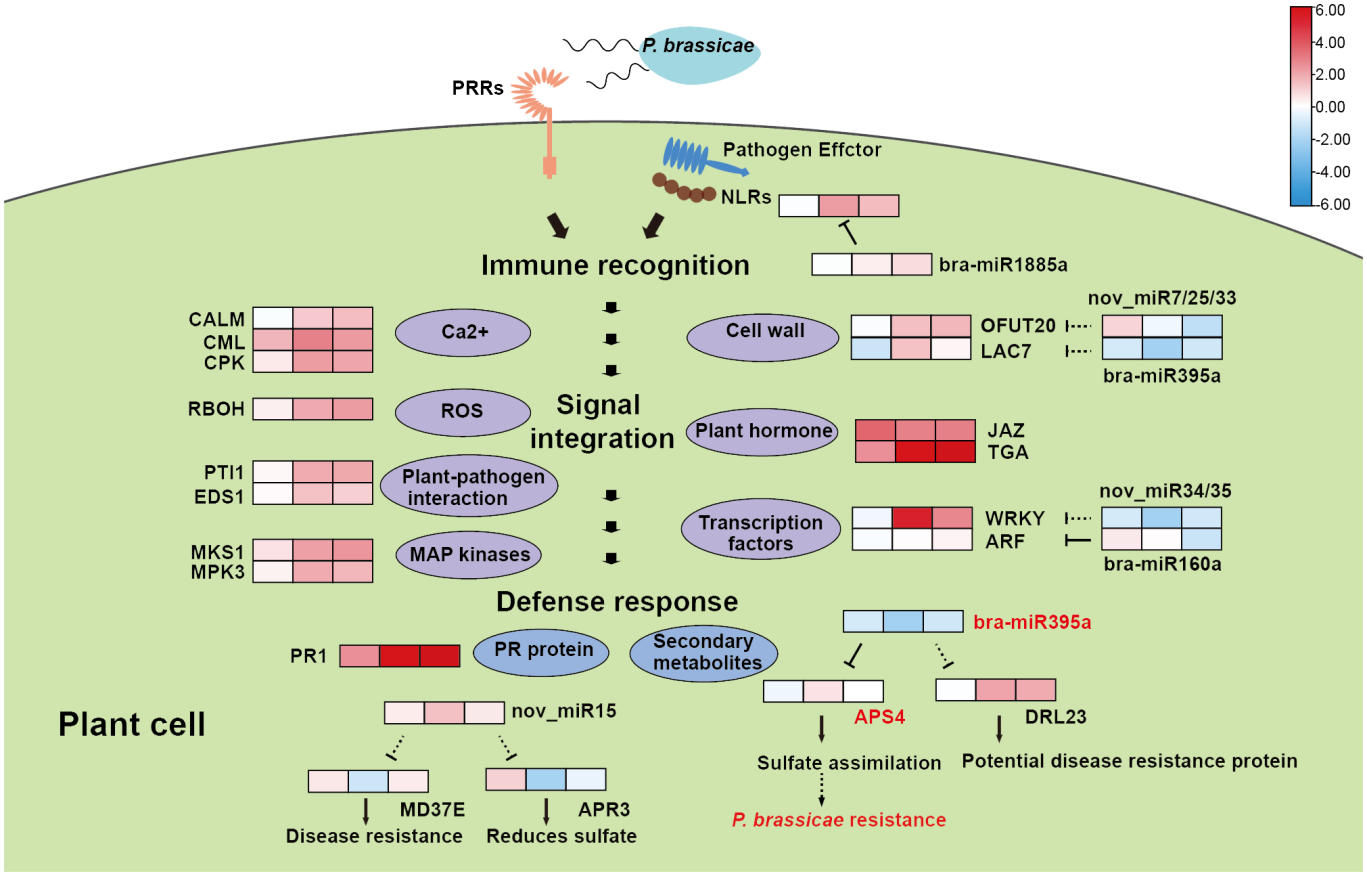
A hypothetical model for the clubroot resistance mechanism in ECD04. The heat maps indicate up-regulation (red) or down-regulation (blue) of gene/miRNA upon infection. Dashed lines represent predicted results and solid lines represent degradome validated results.

In this study, we used RNA-seq, sRNA-seq and degradome to follow the infection procedure of *P. brassicae* in resistant European Turnip ECD04, in order to pinpoint the resistance mechanism to facilitate molecular breeding using key genes and regulators. We have chosen three time points, 18 dpi, 24 dpi and 30 dpi, with uninfected plants as control samples, and identified 318, 224 and 892 DEGs among 20 were shared. Out of these 20 genes, 14 were consistently up-regulated by clubroot infection, therefore they might be positive regulators that are needed for the development of disease resistance. These genes include transcription factors such as *WRKY* and *NAC*, as well as cell wall associated genes, and genes involved in Ca^2+^ influx and signaling (**Figure 7**). GO enrichment analysis found similar results for the DEG sets at 24 dpi and 30 dpi, which is also supported by the WGCNA analysis using all 1751 DEGs. Further, the turquoise module identified from WGCNA revealed good correlation between gene expression and time after infection (**Supplementary Figure 1**), KEGG enrichment of this module also suggested plant-pathogen interaction as the most enriched pathway. From WGCNA we identified four hub genes from the turquoise module (*BraA03.DTX50*, *BraA01.XTH23*, *BraA09.BH019* and *BraA08.TI10A*), by co-expression analysis associated with pathogen-interaction we obtained an additional three hub genes (*BraA07.CDPKS*, *BraA09.CDPKS* and *BraA09.PTI13*). Expression analysis on these 7 genes showed that they were consistently up-regulated throughout 24-30 dpi (**Supplementary Table 2**). Functional annotation revealed that *BraA01.XTH23* participates on cell wall biosynthesis, *BraA09.BH019* is a transcription regulator, *BraA08.TI10A* functions as a repressor of jasmonate responses (Chung and Howe, 2009), while both *BraA07.CDPKS* and *BraA09.CDPKS* are encoding Calcium-dependent protein kinase 28, they are negative immune regulator subjected to ubiquitination and proteasome degradation (Liu et al., 2021). *BraA09.PTI13* is protein kinase that interacts with CLV1 and functions in CLE peptide signaling pathway in root development.

Most of the hub genes from WGCNA analysis are associated with PTI response, yet the defense response in ECD04 most likely occur on both PTI and ETI levels, which all starts from the initial recognition of clubroot pathogen by R proteins. Therefore, we expanded the dataset to include DEGs potentially related to immune response, we identified numerous genes involved in Calcium signaling pathway, formation of reactive oxygen species (ROS), plant-pathogen interaction, MAP kinases, Cell wall related genes, plant hormone and Transcription factors as DEGs upon *Plasmodiophora* infection in ECD04 (**Figure 7**). Some of these genes have been reported to be associated with plant immune response, e.g. *CPK* (Liu et al., 2021), *RBOH* (Wang et al., 2020), *EDS1* (Bhattacharjee et al., 2011), *MPK3* (Meng and Zhang, 2013). Besides, phytohormones including JA and SA, and Calcium influx as well as between cellular comportments are also reported to influence clubroot resistance (Lemarié et al., 2015; Wang et al., 2023). DEGs associated with cell walls might regulate pathogen penetration by increasing the physical barrier capacity by strengthened cell walls. One of the PR genes, *PR1*, was consistently induced at all three time points (18 dpi, 24 dpi and 30 dpi) after clubroot infection, suggesting this gene to be of particular importance. Notably, we also identified NLR genes that were induced in later stages of infection, although not as significantly high levels, indeed these genes might be endogenously regulated to present super induction that might be harmful to the host cell itself. These genes serve as valuable source for future functional validation.

### 4.2 MiRNAs play pivotal role in immune response in ECD04 upon clubroot infection

During recent years, due to the advance in epigenetics and sequencing technologies, people start to realize that small RNAs play important roles as post-transcriptional regulators and could be used for crop genetic improvement (Navarro, 2016; Tang and Chu, 2017; Raza et al., 2023). In the current study, we used high-through-put sequencing and degradome to investigate changes on both transcript levels and microRNAs. We identified 157 known and 52 novel miRNAs, among which 7 were at least differentially expressed at one time point. Indeed, from 18 dpi to 24 dpi and 30 dpi, most DEGs were found at 24 dpi (**Figure 1B**), while the numbers of DEMs rose continuously from 18-30 dpi (**Figure 4C**). Most of the plant miRNAs regulate target gene expression by transcript cleavage, and the cleavage mostly occur at the 10^th^ nucleotide within complementary region. Through target gene annotation, we could deduce the function of microRNAs. And the target genes are obtained normally by two approaches: 1) by software prediction, which predict target genes by sequence complementarity with the mature miRNAs; 2) by degradome sequencing, which requires library construction mediated by miRNA-cleaved fragments. Obviously, parameter settings are also important for target gene filtering. In this study we filtered miRNA-target pairs based on category number (0, 1 and 2) in between mock and inoculation conditions. Consequently, we identified 128 miRNA-target pairs. Within these, the DEM known miRNAs showed miR160-*ARF* and miR395-*APS4* (**Figure 7**), which fits previous study for miR160-*ARF* involved in plant growth and immune response (Yu et al., 2019), and miR395a-*APS4* with sulfite synthesis (Li et al., 2021). For the non-DEMs, we also identified miR164-*NAC* and miR1885a-NLR, these gene pairs have also been reported with HR response (Lee et al., 2017) and R gene expression (Cui et al., 2020). We also used the psRNATarget online prediction to find those miRNA-target pairs with opposite changes upon clubroot infection at three time points. We obtained 15 pairs of miRNA-targets using this approach, including nov_miR7/25/33-*OFUT20*, bra-miR395a-*LAC7*/*DRL23*, nov_miR34/35-*WRKY* and nov_miR15-*APR3*/*MD37E* (**Figure 5A**). Among those, *LAC7* is associated with lignin in cell wall, *APR3* is involved in sulfate assimilation, *MD37E* might play roles in disease response, in addition we also have nov_miR26-*HMA8* which participate in copper-transporting. Therefore, sRNA-seq combined with degradome sequencing could help us to find more reliable miRNA-target pairs that could explain trait phenotypes and provide new viewpoint for genetic breeding.

### 4.3 *Bna-APS4* serves as a negative regulator of clubroot resistance

In comparison with previous studies on microRNA and target pairs, we found our result to be similar with the ones found for miR395a-*APS4* in *B. napus* (Hatzfeld et al., 2000), which also showed down-regulation on miR395 upon clubroot infection in both resistant *B. rapa* or *B. napus* materials and concurrent up-regulation of *APS4*. Combined with our data, we hypothesis that miR395a-*APS4* might play regulatory role in clubroot resistance in *B. rapa* ECD04. Our genetic data on CRISPR-edited *Bna-APS4* showed that loss-of-function mutants in 409S background showed more resistant disease phenotype to its control (409S). This *APS4*-CRISPR result is indeed surprising since it is contradictory to our anticipation for *APS4* as a positive factor for clubroot resistance when miR395a was down-regulated after inoculation (**Table 1**) and *APS4* is the target of miR395a (**Figure 5**). We hypothesize that there might be additional mechanisms of *APS4* that may influence disease outcome, such as the accumulation of sulfate and other metabolites due to *Bna-APS4*-loss-of-function, as reported for *Xoo* infection in rice (Yang et al., 2022b). These hypotheses need to be validated further experimentally. *APS4* is a sulfate adenylyltransferase that catalyze the first step of sulfate assimilation, leading to APS (adenosine 5’-phosphosulfate) from sulfate formation (Mugford et al., 2009). On one side, APS could be reduced back to sulfite to form primary metabolites such as glutathione; on the other side, APS could be an active sulfate form to form secondary metabolites such as glucosinolates (GSH) (Mugford et al., 2009, 2010). These studies showed that some sulfur-containing compounds are also components in the plant immune system, they could function as antioxidants, induce defense gene expression, or limit the growth of pathogenic bacteria (Wang et al., 2022a). For example, GSH is highly relevant for plant immune response, in rapeseed glucosinolate level influence clubroot resistance (Rodrigues and Percival, 2019). In rice, *APS4* mutant results in accumulation of sulfate and elevated resistance towards *Xanthomonas oryzae pv. oryzae* (*Xoo*) and *X. oryzae pv. oryzicola* (*Xoc*) (Rodrigues and Percival, 2019; Zamani-Noor et al., 2021; Yang et al., 2022b). In tomato, cysteine as the key precursor of vascular sulfur accumulation has also been shown to be involved in *Verticillium dahlia* defense (Klug et al., 2014).

We used transcriptome, sRNA-seq and degradome in addition with genetic manipulation of target gene by CRISPR-Cas9 technology, to study the resistant mechanism in ECD04 with superior clubroot resistance. We identified 7 hub genes associated with infection, 15 miRNA-target pairs were predicted and two pairs of particular importance, miR164-*NAC* and miR395a-*APS4*. We validated the function of *APS4* by mutant disease phenotyping. The clubroot-responding miRNA-target network constructed in this study would facilitate further functional studies for some of the hub genes as well as microRNAs, and provide gene candidate to be utilized for clubroot resistance breeding in cruciferous crops in the future.

## Data availability statement

The datasets presented in this study can be found in online repositories. The names of the repository/repositories and accession number(s) can be found in the article/**Supplementary Material**.

## Author contributions

XZ: Conceptualization, Data curation, Writing – original draft, Formal analysis. TZ: Formal analysis, Writing – original draft. MW: Validation, Writing – original draft. QL: Data curation, Writing – review & editing. WY: Formal analysis, Writing – review & editing. LG: Validation, Writing – review & editing. XX: Investigation, Writing – review & editing. YYZ: Data curation, Writing – review & editing. YS: Software, Writing – review & editing. YWZ: Conceptualization, Investigation, Writing – review & editing. PC: Writing – review & editing, Conceptualization, Funding acquisition. CZ: Funding acquisition, Conceptualization, Writing – review & editing.
