# Supplementary material for: Multiomics analysis of a resistant European turnip ECD04 during clubroot infection reveals key hub genes underlying resistance mechanism
Source: Front Plant Sci. 2024 May 23;15:1396602. doi: 10.3389/fpls.2024.1396602 (PMC11153729; doi:10.3389/fpls.2024.1396602)
Supplement: Supplementary file 10 [file DataSheet_1.docx]

Supplementary Material

# Supplementary Data

# Supplementary Figures and Tables

## Supplementary Figures

**
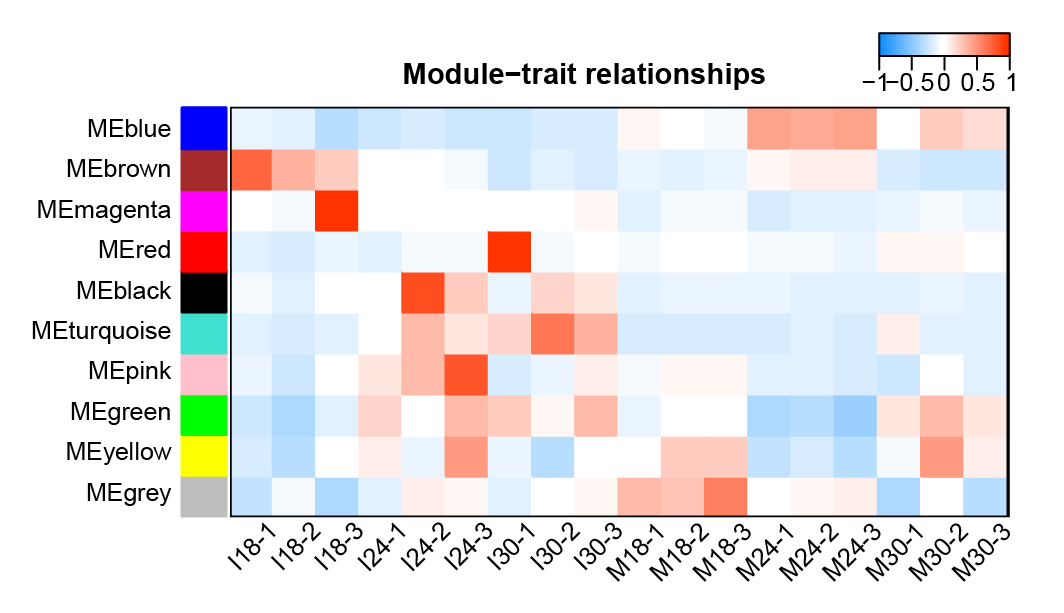
**

**Supplementary Figure 1.** Heatmap of correlation between modules and treatments.

**
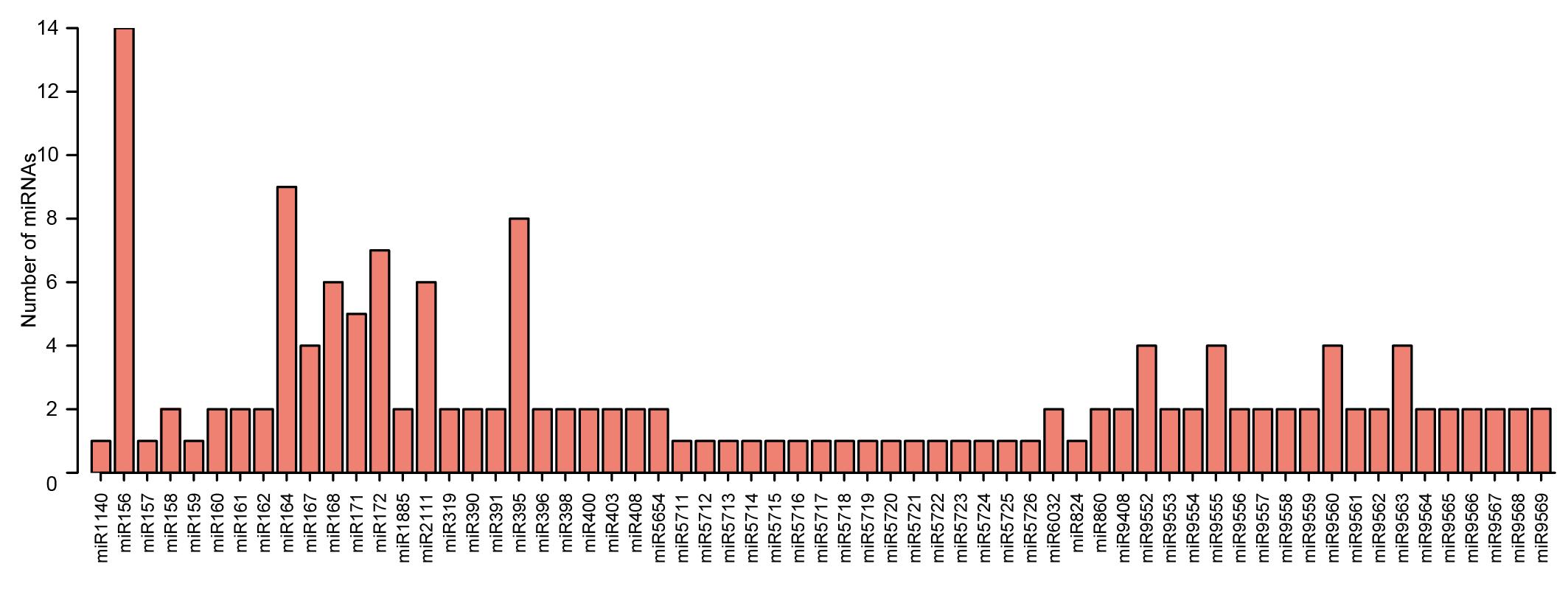
**

**Supplementary Figure 2.** Distribution of known miRNA families through small RNA sequencing.


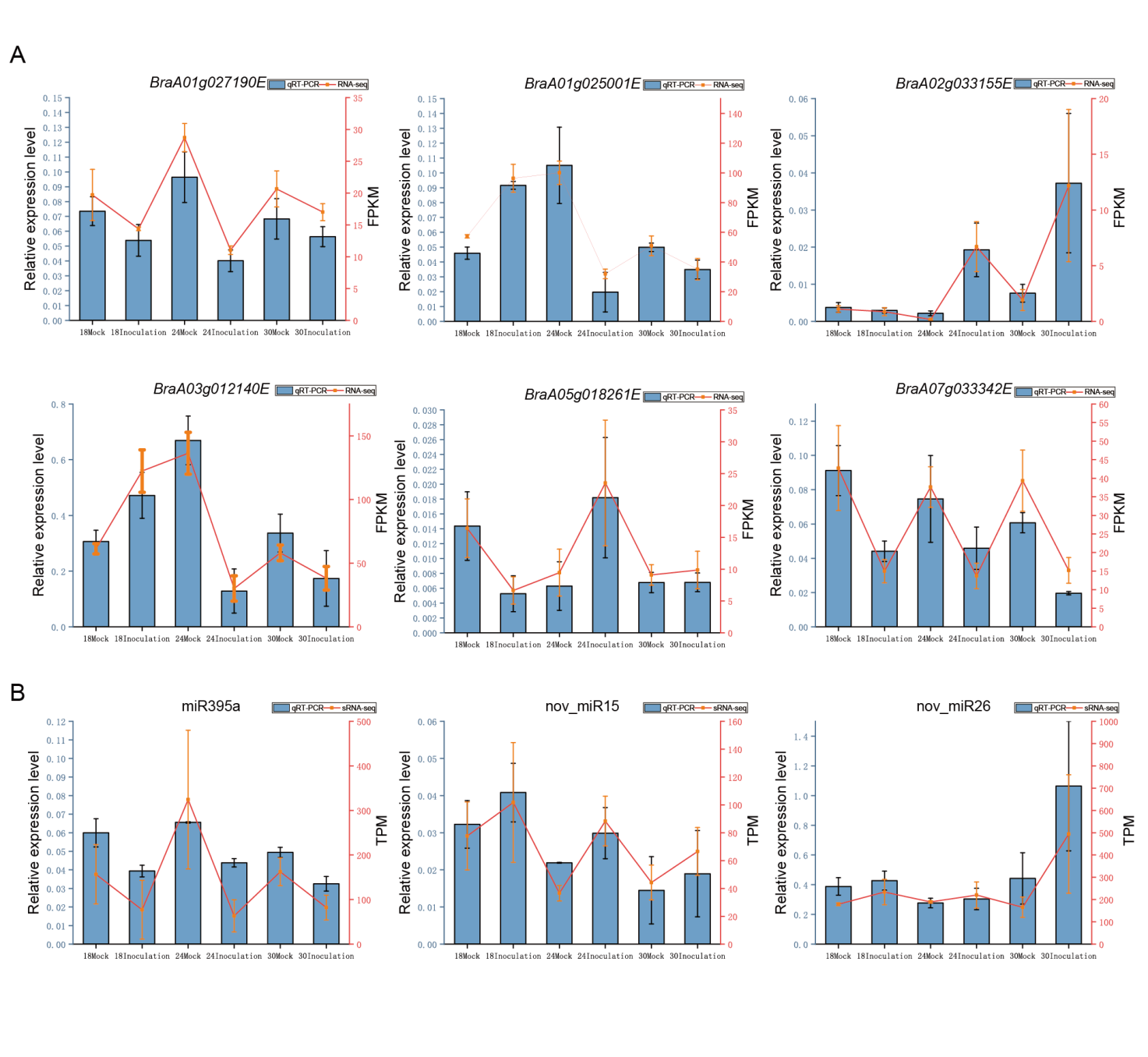


**Supplementary Figure 3.** Validation of DEGs and DEMs expression patterns in ECD04 upon *P. brassicae* inoculation using qRT-PCR. (A) Expression levels of representative DEGs. (B) Expression levels of representative DEMs.


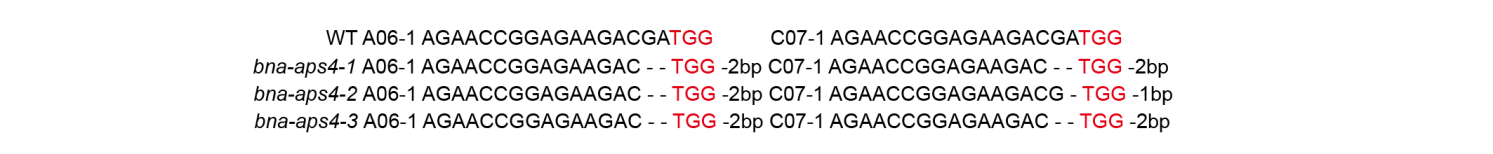


**Supplementary Figure 4.** Sequence alignment of the target region showing deletion in three independent *bna-aps4* mutants. Wild-type (WT, 409S).
